# Bone marrow mesenchymal stem cells transplantation alleviates brain injury after intracerebral hemorrhage in mice through the Hippo signaling pathway

**DOI:** 10.18632/aging.103025

**Published:** 2020-04-09

**Authors:** Xiao Chen, Can-Xin Xu, Huaibin Liang, Zhiyu Xi, Jiaji Pan, Yong Yang, Qingfang Sun, Guoyuan Yang, Yuhao Sun, Liuguan Bian

**Affiliations:** 1 Department of Neurosurgery, Ruijin Hospital, School of Medicine, Shanghai Jiao Tong University, Shanghai 200025, China; 2 Department of Neurology, Ninth People’s Hospital, School of Medicine, Shanghai Jiao Tong University, Shanghai 200011, China; 3 School of Biomedical Engineering and Med-X Research Institute, Shanghai Jiao Tong University, Shanghai 200030, China; 4 Department of Neurosurgery, Guangdong Provincial People’s Hospital, Guangdong Academy of Medical Sciences, Guangzhou 510080, China

**Keywords:** ICH, MSC, astrocyte, Hippo, YAP

## Abstract

Intracerebral hemorrhage (ICH) is a common acute nervous system disease with high mortality and severe disability. Mesenchymal stem cells (MSCs) have been reported to promote neurogenesis and to alleviate side effects in areas of brain injury areas. The Hippo pathway regulates diverse cellular processes, including cell survival, proliferation, differentiation, and organ size. Here, we found that transplantation of bone marrow MSCs (BM-MSCs) into the brains of mice could alleviate ICH-mediated injury and protect astrocytes from apoptosis by regulating mammalian sterile 20-like kinase (MST)1 and Yes-associated protein (YAP). Knocking down of MST1 by si-RNA triggered YAP nuclear translocation. We further demonstrated that astrocytes undergo astroglial-mesenchymal phenotype switching and become capable of proliferating after BM-MSC transplantation via the Hippo signaling pathway. Together, our identification of the Hippo pathway in mediating the beneficial effects of BM-MSCs may provide a novel therapeutic target in the treatment and management of ICH.

## INTRODUCTION

Intracerebral hemorrhage (ICH), which is characterized by high mortality and disability, is the most severe form of stroke with no effective treatment modality, although considerable progress has been made in animal and preclinical research [1, 2]. ICH often comprises a series of injuries caused by two different phases. The formation and expansion of ICH results in mechanical damage of the brain tissue, followed by edema, inflammation, and necrosis, which lead to the destruction of neurons and glial structures, an aberrant release of the neurotransmitters and mitochondrial dysfunction, and ultimately to apoptosis. In the central nervous system (CNS), astrocytes constitute one of several types of glial cells, which provide not only structural support but also nutritional supply for neurons and help maintain homeostasis of the extracellular environment [3, 4]. In response to the CNS is threatened and damaged, such as nerve injury, infection, ischemia, hemorrhagic stroke, or neurodegeneration, astrocytes are activated, which causes them to proliferate, migrate, and finally to form glial scars [5]. A better understanding of the potential mechanisms underlying astrocyte activation may help to improve the prognosis of patients.

Mesenchymal stem cells (MSCs) are the prototype of pluripotent stem cells and have potential applications in regenerative medicine. MSCs are self-renewing and multipotent cells that generate progenitors, which are capable to differentiate into various and distinct cell lineages. They can be isolated from different human tissues, amplified and/or differentiated *in vitro*, and then used for the treatment of various diseases. The mechanism by which MSCs exert their regenerative activity is mainly related to their nutritional effects on other cells, as well as their highly anti-inflammatory and immunomodulatory capabilities [6, 7]. Jiang et al. have revealed that bone marrow MSCs (BM-MSCs) could interact with the injured microenvironment, and shift the balance from a toxic to a protective and regenerative milieu via releasing bioactive factors [8]. BM-MSCs were also found to promote neurogenesis and alleviate late side effects in brain injury areas, including an enhancement of the vascular system and restoration of motor, sensory, and cognitive functions [9]. These specific characteristics make BM-MSCs a promising therapeutic modality for several CNS conditions, such as ischemic stroke and ICH [10, 11].

The Hippo pathway is evolutionally conserved and regulates a myriad of cellular processes, including cell survival, proliferation, differentiation, and organ size. In mammals, this Hippo pathway consists of the serine/threonine kinases mammalian sterile 20-like kinase 1/2 (MST1/2) and large tumor suppressor 1/2 (LATS1/2) [12], while Yes-associated protein (YAP) is its major downstream mediators of the Hippo pathway. Activation of the Hippo pathway results in the inactivation of YAP by LATS1/2-mediated direct phosphorylation. Phosphorylated YAP is sequestered in the cytoplasm by binding to 14-3-3 and subsequently degraded in a ubiquitin-proteasome-dependent manner [13]. Conversely, dephosphorylation of YAP induces its transport into the nucleus and subsequent interaction with TEA/ATTS domain (TEAD), forkhead box protein O1 (FoxO1), and other transcription factors, thereby inducing cell proliferation, organ growth, stem cell self-renewal, epithelial-mesenchymal transition, and inhibition of apoptotic gene expression [12–14]. Zhao et al. advanced an additional consideration, namely that the Hippo pathway functions not only in cancer cells but also in immune cells [15]. Inflammatory conditions could affect the expression of MST1, further promoting inflammation, fibrosis, and tumorigenesis [16, 17]. In the CNS, MST1 was also found to be involved in neuro-inflammation caused by cerebral ischemia [18]. Knockdown of MST1 was shown to reduce neuronal death and ameliorate neurological impairment in traumatic brain injury [19]. The YAP signaling pathway has also been also implicated in astrogliogenesis and astrocytic differentiation in the developing neocortex [20], and YAP knockout mice develop reactive astrogliosis in the cortex, supporting its critical involvement in brain development [21]. In our previous studies, we have found that astrocytes undergo dedifferentiation via Hippo signaling pathway following ICH [4].

Based on these foundations, we aimed to elucidate whether BM-MSCs could alleviate brain injury and promote astrocyte proliferation via the Hippo signaling pathway. We used both *in vivo* and *in vitro* techniques to test our hypothesis.

## RESULTS

### BM-MSCs purification and identification

BM-MSCs were successfully isolated from the femoral and tibial bone marrow of adult male Sprague-Dawley rats and cultured in the medium for several generations. The cultured cells demonstrated a typical spindle-shaped morphology (Supplementary Figure 1A, 1B). Flow cytometry analysis confirmed that the cells used for transplantation experiments were positive for CD29 (99.83%) and CD90 (99.87%), and had low expression of CD34 (0.46%) and CD45 (1.33%) (Supplementary Figure 1C), which is highly consistent with the previous publications [22]. Using a red fluorescent dye (PKH-26), we were able to track positive transplanted cells, which were confirmed to be located in the ICH hemisphere 3d after transplantation (Supplementary Figure 1D, 1E).

### BM-MSCs transplantation reduced hematoma volume and alleviated neurological deficits after ICH

**Figure 1:**
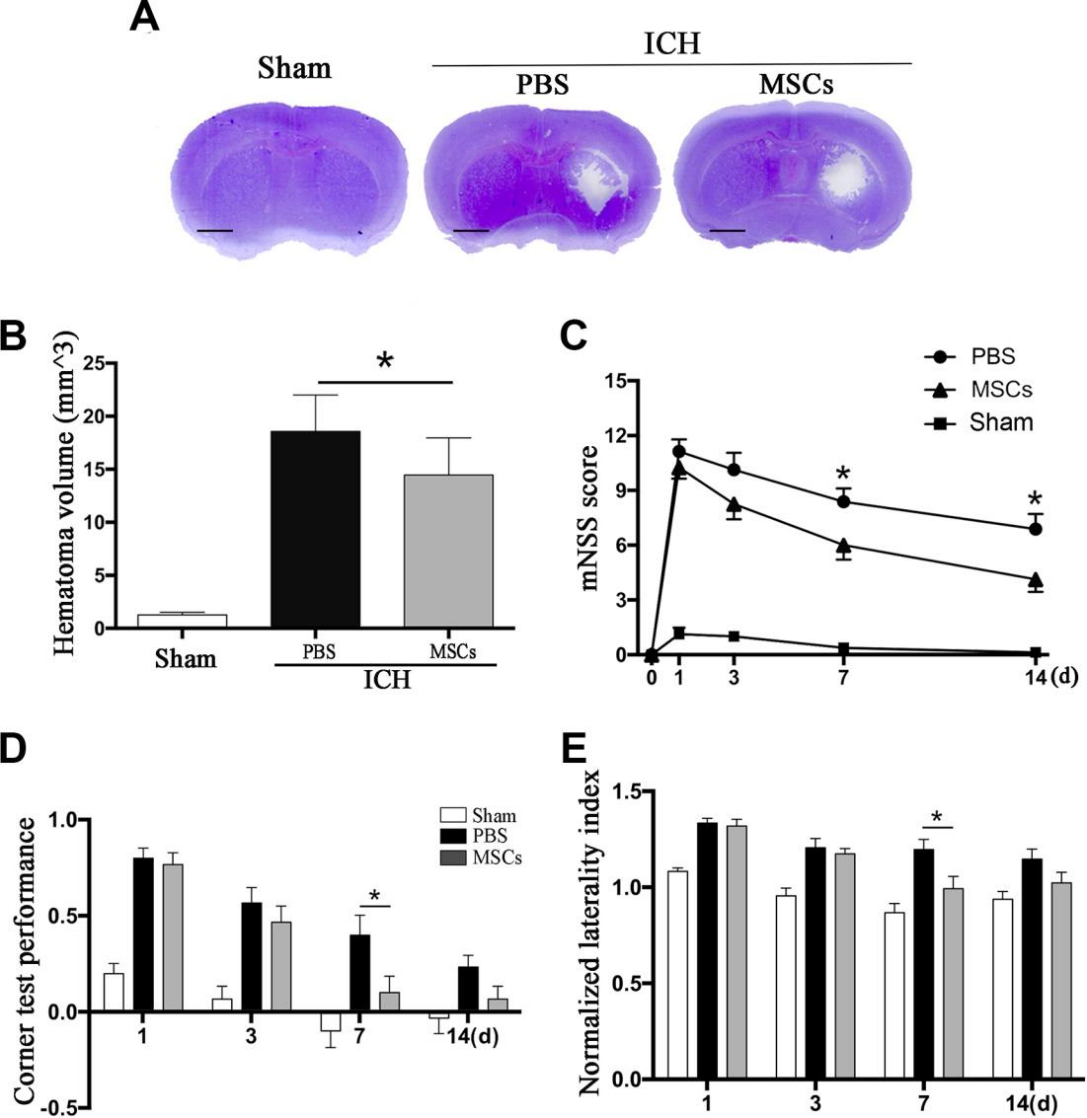
**BM-MSCs transplantation reduced hematoma volume and improved neurological outcomes after ICH.** (**A**, **B**) The volume of ICH in BM-MSCs and PBS treated Mice after 3 d post-transplantation with Cresyl violet staining. Bar = 1mm. (**C**) BM-MSCs improved neurological outcomes both in mNSS and corner test. All data are displayed as means ± SD (n = 10). (**C**–**E**) *p< 0.05, compared with the PBS group.

BM-MSCs transplantation led to a decrease in ICH hematoma volume on day 3 as compared with the PBS group (*p* < 0.05) (Figure 1A). We further tested the neurological outcomes at 1, 3, 7 and 14d after MSCs transplantation using the modified neurological severity score (mNSS) and the corner test. We observed a significant improvement in neurological deficits after BM-MSCs transplantation at 7 and 14d post-procedure (*p* < 0.05) (Figure 1B–1E), compared with the PBS group.

### BM-MSCs transplantation promoted VIM, EAAT1, and YAP expression in astrocytes after ICH *in vivo*

**Figure 2:**
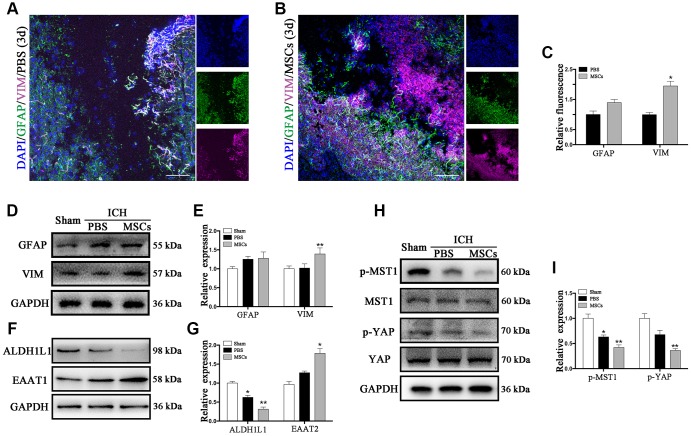
**The astroglial mesenchymal phenotype switching of astrocytes in ICH.** (**A**) Immunofluorescence staining for VIM (purple) and GFAP (green) in ICH mouse brain at 3d post-PBS transplantation. Bar = 100μm. GFAP and VIM were expressed in reactive astrocytes around the lesion area. (**B**) Immunofluorescence staining for VIM (purple) and GFAP (green) in the ICH mouse brain at 3d post-BM-MSCs transplantation. Bar = 100μm. After the transplantation of BM-MSCs, VIM was strongly expressed, whereas GFAP remained at the same level. (**C**) The results of relative fluorescence intensity of GFAP and VIM, plotting into a histogram of five randomly fields. (**D**–**I**) Western blotting analysis of GFAP, VIM, ALDH1L1, EAAT1, p-MST1, MST1, p-YAP and YAP expression in the ICH mouse brain of Sham, PBS, and BM-MSCs treatment at 3d. Expressions were normalized against the internal reference GAPDH. The fold change values were calculated by normalizing to the sham group. The results were plotted as mean ± SD (n = 6). *p < 0.05, compared with sham group.

Vimentin (VIM) and glial fibrillary acidic protein (GFAP) are the two kinds of intermediate filaments (IFs) associated with astrocytes activation and reactive gliosis, which were shown to be expressed in the early and late stages of CNS damage. Therefore, the increased expression of VIM and GFAP are widely regarded as markers of reactive astrocytes [23, 24]. Immunofluorescence staining and Western blot were applied to detect the expression of these proteins following ICH. As shown in Figure 2A, VIM and GFAP were expressed at the hematoma margin in the PBS group after ICH at 3d post-procedure. Following BM-MSCs transplantation, the expression of VIM significantly increased, whereas the observed increase in GFAP was not statistically significant (Figure 2A–2C), which was confirmed by Western blot (*p* < 0.05) (Figure 2D, 2E). We further examined the protein expression of aldehyde dehydrogenase 1 family member L1 (ALDH1L1) and excitatory amino acid transporter 1 (EAAT1). After BM-MSCs transplantation, the expression of ALDH1L1 decreased (*p* < 0.01), while the expression of EAAT1 increased (*p* < 0.05) (Figure 2F, 2G). In addition, the expression of phosphorylated (p-) MST1 and p-YAP, which decreased after ICH, decreased further following BM-MSCs transplantation, compared to the sham group (*p* < 0.05) (Figure 2H, 2I).

### BM-MSC-astrocyte co-culture enhanced astrocytes resistance of astrocytes to hemin neurotoxicity, promoted their proliferation, regulated cytokine mRNA expression, and increased TEAD2 expression *in vitro*

**Figure 3:**
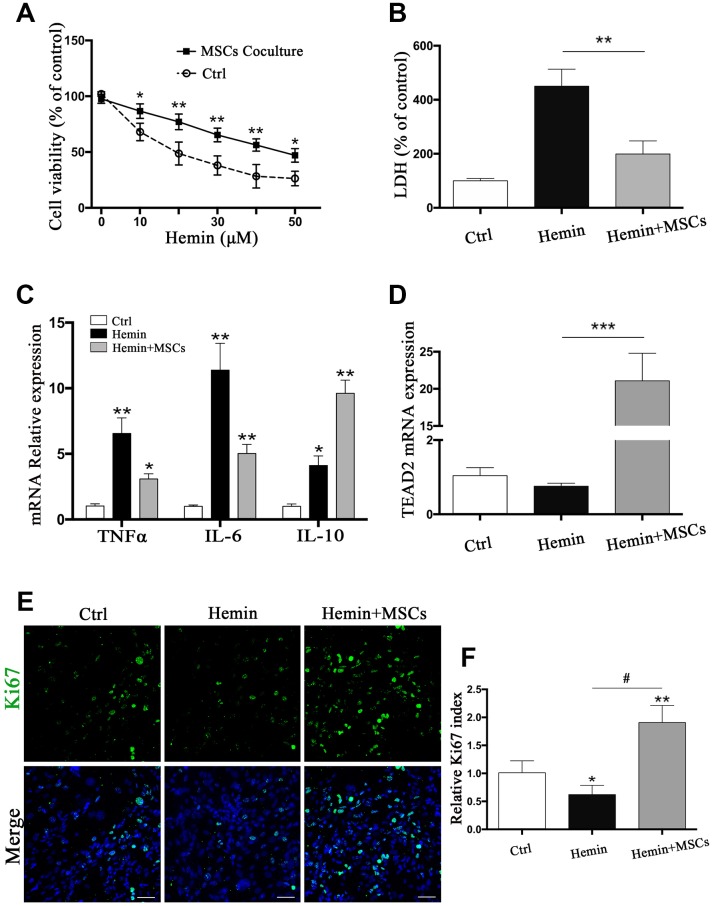
**BM-MSCs coculture protected astrocytes from neurotoxicity induced by hemin.** (**A**) Astrocytes were exposed to 0, 5, 10, 20, 30, 40, 50μM hemin for 24 h with or without BM-MSCs coculture, then the cell viability was evaluated by CCK-8. (**B**) Astrocytes were exposed to 30μM hemin with or without BM-MSCs for 24 h, and the cell death was evaluated by LDH releasing assay; (**C**) mRNA expression of TNFα, IL-6, and IL-10 was checked. (**D**) mRNA expression of TEAD2 was checked. (**E**, **F**) Ki67 staining (green) was applied to mark cell proliferation, bar = 25μm. The results plotted as mean ± SD (n = 4), and the relative expression of the mRNA and results of proliferation rate were normalized to control and plotted into a histogram. *p< 0.05, **p < 0.01, ***p < 0.001 compared with ctrl; #*p* < 0.001.

Given our findings that astrocytes responded to BM-MSCs in our *in vivo* experiments, so we established an ICH model *in vitro* by exposing primary astrocytes to hemin, with or without BM-MSCs coculture, as shown in Supplementary Figure 1A. We assessed astrocytes viability and death by the cholecystokinin (CCK)-8 and lactate dehydrogenase (LDH) releasing assays after hemin exposure with or without BM-MSCs (at a ratio of 1:10). The results showed that astrocyte viability decreased in hemin dose-dependent manner, and subsequent experiments were carried out with 30 μM hemin as this concentration significantly increased cell mortality (*p* < 0.01) (Figure 3A, 3B). Expression of the cell cycle marker Ki67 was analyzed by immunofluorescence staining to determine whether BM-MSCs could promote astrocyte proliferation. As shown in Figure 3E, 3F, the percentage of Ki67-positive astrocytes significantly increased when co-cultured with BM-MSCs (*p* < 0.01). Using quantitative real-time polymerase chain reaction (qPCR) we further found that mRNA expression of tumor necrosis factor (TNF)α and interleukin (IL)-6 significantly decreased upon co-culture with BM-MSCs (*p* < 0.05 for both), while IL-10 significantly increased, as compared to astrocyte monoculture (*p* < 0.05) (Figure 3C). Astrocytic TEAD2 mRNA levels also significantly increased in the presence of BM-MSCs in comparison with monocultures (*p* < 0.0001) (Figure 3D). These results support the ability of BM-MSCs to activate astrocytes and enhance their proliferation and resistance to hemin neurotoxicity.

### BM-MSC-astrocyte co-culture induced an astrocyte-mesenchymal phenotype *in vitro*

To confirm BM-MSC-mediated activation of astrocytes *in vitro*, immunofluorescence staining was performed to detect astrocytic GFAP and VIM in astrocytes with or without BM-MSCs co-cultures.

**Figure 4:**
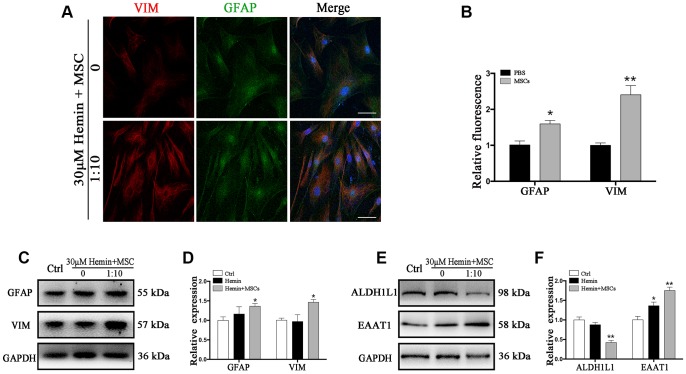
**BM-MSCs coculture induced an epithelial-mesenchymal switching in astrocytes.** (**A**) Immunofluorescence staining of astrocytes for VIM (red) and GFAP (green). The cell nuclei were counterstained with DAPI (blue). Bar = 25 μm. (**B**) The results of relative fluorescence intensity of GFAP and VIM, plotting into a histogram of five randomly fields. (**C**–**F**) Western blotting analysis of GFAP, VIM, ALDH1L1, and EAAT1 in astrocytes exposure to 30 μM hemin, with or without BM-MSCs coculture for 24 h. BM-MSCs coculture increased VIM, GFAP and EAAT1 expression, while decreasing ALDH1L1 expression. Expressions were normalized against the internal reference GAPDH. The fold change values were calculated by normalizing to control samples. The results were plotted as mean ± SD (n = 6). *p < 0.05, **p < 0.01 compared with ctrl group.

We indeed observed an increase in VIM expression in astrocytes (Figure 4A, 4B), and Western blot corroborated increased VIM and GFAP expression in the presence of BM-MSCs compared to monocultures (*p* < 0.05) (Figure 4C, 4D). Additionally, the expression of ALDH1L1 significantly decreased, while EAAT1 levels increased in co-cultures compared to astrocyte monocultures (*p* < 0.01 for both) (Figure 4E, 4F). Together, our observations confirm that BM-MSCs induce a shift in astrocytes toward an astroglial-mesenchymal phenotype *in vitro*.

### BM-MSC-astrocyte co-culture restrained MST1 phosphorylation and induced YAP nuclear translocation *in vitro*

**Figure 5:**
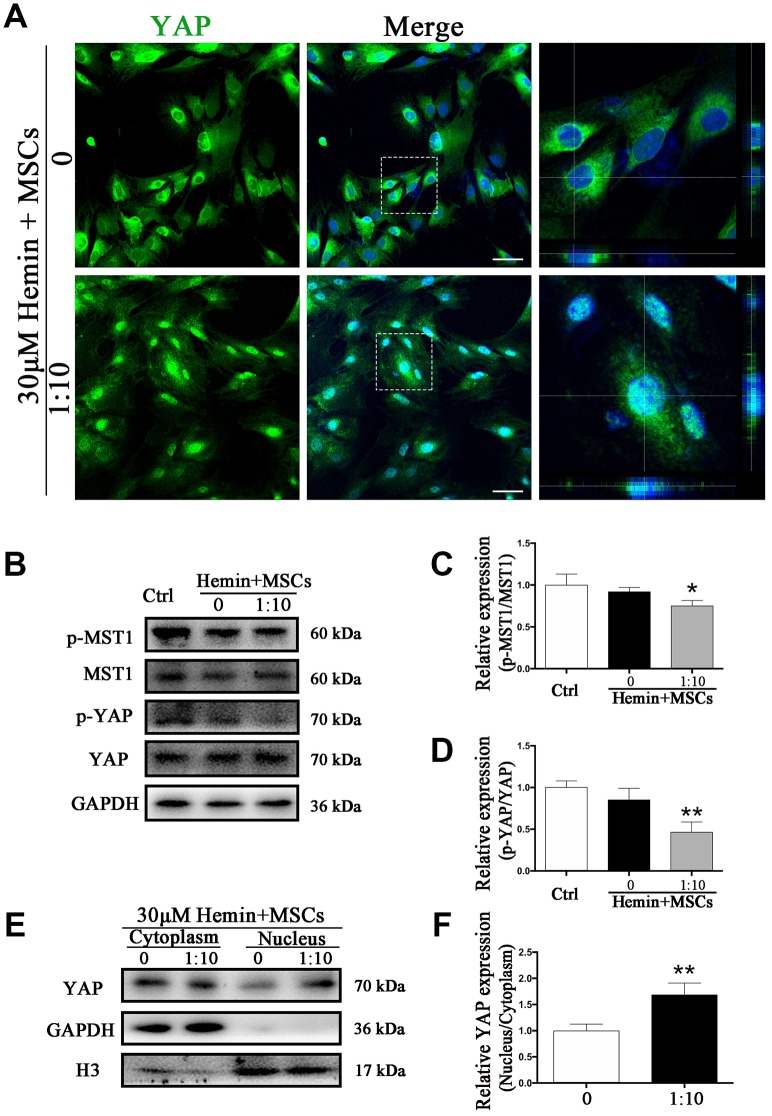
**BM-MSCs coculture promoted YAP nuclear translocation in astrocytes.** (**A**) Immunofluorescence staining of astrocytes with YAP (green). The cell nuclei were counterstained with DAPI (blue). BM-MSCs coculture treatment triggered YAP nuclear translocation in astrocytes exposure to 30 μM hemin. Bar = 25 μm. (**B**–**D**) Western blotting analysis of p-MST1, MST1, p-YAP and YAP expression in astrocytes exposure to 30 μM hemin, with or without BM-MSCs coculture for 24 h. (**E**, **F**) Western blotting analysis of cytoplasmic and nucleus extraction samples from astrocytes, with or without BM-MSCs coculture, with YAP antibody. GAPDH and H3 were used as a loading control for cytoplasmic and nucleus protein, respectively. The histogram showing the results of densitometric analysis of nucleus/cytoplasmic YAP expression in astrocytes cocultured with BM-MSCs or not. The results were normalized to control and plotted as mean ± SD (n = 4). *p < 0.05, **p < 0.01, compared with ctrl group.

To test whether phosphorylation levels of MST1 and YAP in astrocytes may be affected by BM-MSCs we performed Western blot analysis of p-MST1, MST1, p-YAP, and YAP from BM-MSC-astrocyte co-cultures. The results show that the ratios of p-MST1/MST1 and p-YAP/YAP were significantly decreased as compared with astrocyte monocultures (*p* < 0.05, *p* < 0.01, respectively) (Figure 5B–5D). In addition, immunofluorescence staining demonstrated that hemin exposure triggered more YAP nuclear translocation in astrocytes co-cultured with BM-MSCs compared to those in monoculture (Figure 5A). This was also confirmed by significantly higher nuclear to cytoplasmic ratios of YAP expression in astrocytes in the presence of BM-MSCs as compared with astrocyte monoculture (*p* < 0.01) (Figure 5E, 5F).

### Knockdown of YAP attenuated BM-MSC-induced astrocyte proliferation

**Figure 6:**
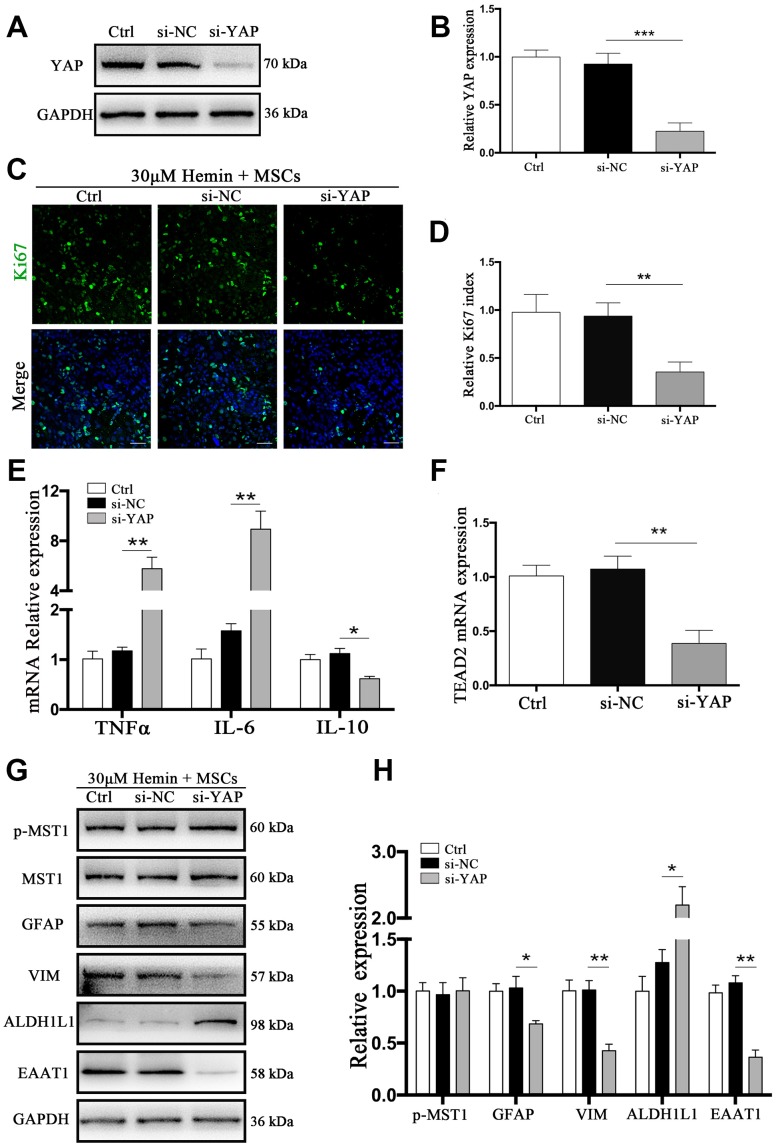
**si-YAP counteracted BM-MSCs coculture induced cell proliferation and GFAP/VIM switching.** (**A**) Western blotting analysis of YAP expression in ctrl, si-NC, and si-Nrf2 transfected astrocytes. (**B**) The results of densitometric analysis of the bands were plotted as mean ± SD (n = 4). Over 83% of YAP expression was suppressed by si-YAP. ***p < 0.001 compared with si-NC group. (**C**) Representative pictures of Ki67 immunofluorescence staining. Ki67-positive cells were labeled in green. Bar = 50μm. (**D**) The results of the proliferation rate and densitometric analysis of the bands were plotted into a histogram of five randomly fields. (**E**). mRNA expression of TNFα, IL-6, and IL-10 was checked. (**F**) mRNA expression of TEAD2 was checked. (**G**, **H**) Western blotting analysis of p-MST1, MST1, GFAP, VIM, ALDH1L1, and EAAT1 protein expression was examined. The relative expression was normalized to control. The results of densitometric analysis of the bands were plotted as mean ± SD (n = 4). *p < 0.05, **p < 0.01.

YAP has transcriptional activity upon translocation into the nucleus, interacting with TEAD, FoxO1, and other transcription factors to promote cell proliferation and epithelial-mesenchymal transition, and to inhibit apoptosis [12]. We used small interfering RNA (si-RNA) specific to YAP (si-YAP) to knock down its expression by more than 83% (*p* < 0.001) (Figure 6A, 6B). The proportion of Ki67-positive astrocytes co-cultured with BM-MSCs was significantly decreased by si-YAP compared with si-NC (*p* < 0.01) (Figure 6C, 6D). Cytokine and TEAD2 mRNA expression was reversed through YAP knockdown in BM-MSC-astrocyte co-cultures, whereby TNFα and IL-6 increased (*p* < 0.01 for both), while IL-10 decreased, as compared to si-NC (*p* < 0.05) (Figure 6E). TEAD2 mRNA levels significantly decreased (*p* < 0.01) (Figure 6F). Furthermore, si-YAP reversed the previously observed BM-MSC-mediated upregulation of VIM and EAAT1 (*p* < 0.05, *p* < 0.01 respectively), and caused a significant increase in ALDH1L1 as compared to the si-NC group (*p* <0.05) (Figure 6G, 6H). These data support the role of YAP in the proliferation and mesenchymal phenotype transition in astrocytes via BM-MSCs.

### Knockdown of MST1 triggered YAP nuclear translocation in astrocytes

**Figure 7:**
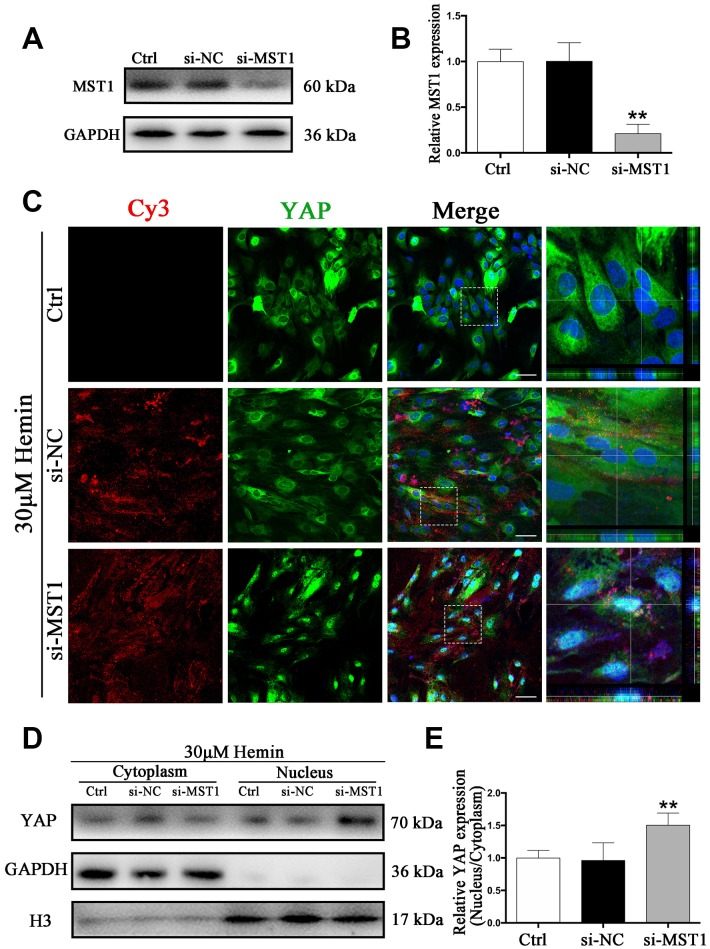
**Knockdown of MST1 triggered YAP nuclear translocation.** (**A**) Western blotting analysis of MST1 expression in ctrl, si-NC, and si-Nrf2 transfected astrocytes. (**B**) The results of densitometric analysis of the bands were plotted as mean ± SD (n = 4). Over 80% of MST1 expression was suppressed by si-MST1. **p < 0.01 compared with ctrl group. (**C**) Immunofluorescence staining of ctrl, si-NC, si-MST1 treated astrocytes with anti-YAP (green). The siRNA was labeled with fluorophore Cy3 (red) to show the transfected cells. The cell nuclei were counterstained with DAPI (blue). Bar = 50μm. (**D**, **E**) Western blotting analysis of cytoplasmic and nucleus extraction samples from control, si-NC, si-MST1 transfected astrocytes with anti-YAP. GAPDH and H3 were used as a loading control for cytoplasmic and nucleus protein, respectively. The histogram showing the results of densitometric analysis of nucleus/cytoplasmic YAP expression in ctrl, si-NC, si-MST1 transfected astrocytes. The results were normalized to control plotted as mean ± SD (n = 4). Si-MST1 significantly increased YAP nuclear expression. *p < 0.01 compared with control.

We next sought to explore the mechanism of the Hippo signaling pathway in ICH. Specifically, we wanted to know whether YAP nuclear translocation may be triggered by MST1 downregulation. To this end, we used MST1-si-RNA (si-MST1), which caused a reduction in MST1 expression by more than 80% (*p* < 0.01) (Figure 7A, 7B). As shown by immunofluorescence staining, YAP nuclear translocation was evident in si-MST1 transfected astrocytes, while in the control and si-NC transfected group YAP was mainly located in the cytoplasm (Figure 7C). These results could be corroborated by Western blot analysis (Figure 7D, 7E). Our findings confirm our hypothesis that MST1 downregulation triggers YAP nuclear translocation in astrocytes, and the proposed mechanism is shown in Supplementary Figure 2D.

## DISCUSSION

Astrocytes are one type of main glial cells in the CNS and play key roles in maintaining CNS homeostasis, including neurotrophic and structural supporting functions, maintaining the extracellular environment, stabilizing intercellular communications, and regulating oxidative stress in this organ [25]. ICH is characterized by a series of pathological events, such as increased inflammation and cell death around injured perihematomal tissues. Astrocyte activation is a common response to CNS damage and reactive astrocytes can produce neurotrophic factors, protect neurons from injury, isolate injured sites from healthy tissues by forming a physical and chemical barrier, and prevent waves of harmful inflammation. On the other hand, reactive astrocytes are also known to inhibit axonal regeneration and obstruct other repair processes within the CNS [26, 27], rendering them a potential target in the treatment of ICH, whereby their beneficial effects could be enhanced and their detrimental effects limited. Astrocytes of VIM-GFAP double-knockout mice are deficient in cytoplasmic IFs, with the consequence of decreased reactive glial degeneration and lack of characteristic hypertrophy of astrocyte processes after CNS injury [28]. In our previous studies, we have proposed that astrocytes may undergo dedifferentiation following ICH [4], which could be a stringent mechanism for the cells in response to injury, promoting their activation and survival. VIM is essential for proper cell spreading, which could regulate adhesion, adhesion strength, and focal contact size, thereby controlling the isolation of injured sites from healthy tissues [23, 29].

MSCs, when transplanted, are known to pass through the blood brain barrier, migrate to sites of injury and inhibit apoptosis of astrocytes [11, 30]. Early and sustained beneficial effects of MSCs secreting bioactive factors with neurotrophic/immunoregulatory potential in CNS injury have been observed [31]. In the present study, we found that transplantation of BM-MSCs into the CNS of mice with ICH significantly improved cognitive and motor function and reduced hemorrhagic volume, which is in line with previous studies [9]. MSCs therapy has also been reported to modulate microglia activation by maintaining a resting, regenerative microglial phenotype, or by limiting the microglial activation after stroke [32, 33]. Remarkably, Donega et al. also reported that MSCs treatment successfully reduced GFAP expression and glial scars formation in humans [34].

Here, we observed that BM-MSC transplantation resulted in an elevation of VIM and GFAP expression, even though the change of the latter was not statistically significant. Both proteins were significantly increased in BM-MSC-astrocyte co-cultures, supporting our *in vivo* findings.

As VIM is not astrocyte-specific but is expressed in many cell types, we further examined the expression of ALDH1L1 and EAAT1. ALDH1L1 is expressed in most quiescent cells in the developing mouse brain, while proliferating cells do not express this protein [35]. It is an abundant cytosolic enzyme involved in folate pathways, essential for several major cellular processes including biosynthesis of precursors for DNA and RNA [36]. Since the activation and proliferation of astrocytes in co-culture with BM-MSCs would lead to the consumption of folate, this may explain why they showed lower ALDH1L1 expression as compared to monocultures. EAAT1, which was localized in areas of developing glial scar, was increased after ICH, and this increase was amplified through BM-MSCs both *in vivo* and *in vitro*. The majority of adult astrocytes are terminally differentiated, although under certain conditions, they may be able to divide or even display stem cell-like behavior to prevent damage or recover from injury [37]. This could be one possible explanation for the higher expression of VIM and EAAT1 by astrocytes in our experiments. In addition, we show that astrocytes co-cultured with BM-MSCs were protected from neurotoxicity *in vitro*, which was accompanied by a downregulation of the pro-inflammatory cytokines TNFα and IL-6 and an upregulation of the anti-inflammatory cytokine IL-10; this is consistent with the results of a previous report by Aggarwal and colleagues [38].

The Hippo pathway is a key regulator of tissue homeostasis, mediating contact inhibition signaling and regulating organ size by controlling cell proliferation and expansion. It has been reported that YAP is selectively expressed in neural stem cells (NSCs) and astrocytes, but not neurons [39]. In astrocytes, YAP is required for astrocytic proliferation, and the deletion of this protein in NSCs or astrocytes leads to impaired astrogliogenesis and increased neocortical neurodegeneration [20]. Repression of either YAP or TEADs in the neural tube causes a significant increase in cell death, cell cycle exit, and differentiation of neuronal precursors [39].

In our *in vivo* experiments, p-MST1 and p-YAP were highly expressed in the normal brain, and nuclear translocation of YAP was suppressed. Upon ICH, the levels of p-MST1 and p-YAP decreased, and following BM-MSC transplantation, YAP was translocated into the nucleus. Similar results were found in co-cultures *in vitro*, whereby BM-MSCs inhibited phosphorylation of MST1 and YAP, triggering their nuclear translocation in astrocytes. In addition, TEAD2 mRNA expression was increased in astrocytes in the presence of BM-MSCs. Subsequently, astrocyte dedifferentiation enhanced and VIM expression increased, which inhibited apoptosis, promoted proliferation, and ultimately reduced hemin-mediated neurotoxicity. Conversely, we found that astrocyte proliferation and dedifferentiation was inhibited upon YAP knockdown *in vitro*. High YAP activity enables the cell to circumvent contact inhibition, induces epithelial-mesenchymal transition, and eventually tumor development [40].

ALDH1L1, on the other hand, is silent in malignant tumors, and its re-expression in cancer cells elicits anti-proliferative effects [35, 41]. This may explain our finding that ALDH1L1 was highly expressed in astrocytes following YAP knockdown, and that BM-MSC-induced astrocyte proliferation was attenuated due to the subsequent blockage of YAP-TEAD interactions.

On the other hand, knockdown of MST1 induced YAP dephosphorylation triggering their nuclear translocation. Mo et al. have previously shown that a loss of MST1/2 or LATS1/2, or activation of YAP-TEAD led to a marked expansion of neural progenitors [14]. Chen and Yuan et al. have reported that inhibition of MST1 or reduction in p-MST1 level could effectively alleviate neurological deficits during cerebral ischemia-reperfusion injury and ICH [42, 43]. However, MST1 was also found to activate inflammatory cytokines such as TNF-α and IL-6, which caused inflammation and cell death [42], while BM-MSCs could regulate the production of inflammatory cytokines [38]. Taken together, our findings, in support by previous studies, suggest that transplanted BM-MSCs ameliorated neurological deficits in ICH mice via inhibiting the MST1/Hippo pathway in astrocytes.

There are several limitations to our study. Firstly, although we used si-RNA to interfere with MST1, it is ineffective for other members of the MST family, which may have affected our results. Secondly, MST is only one of the upstream regulatory factors of YAP, and others such as LATS were not investigated in this study. Lastly, we only analyzed changes during the early stage of ICH, so that further research is needed to explore the effect of BM-MSCs in later stages of this condition.

In conclusion, we demonstrated that BM-MSC transplantation has an important role in alleviating neurological deficits, promoting astrocyte proliferation and mesenchymal phenotype switching after ICH. These effects were at least in part mediated via BM-MSC-mediated regulation of the MST1/YAP/Hippo signaling pathway, thus supporting the potential clinical value of YAP as a target in the treatment of brain injury after ICH.

## MATERIALS AND METHODS

### Animals and ethics

Adult male C57BL/6 mice aged 6 - 8 weeks, weighing 22 - 25g were purchased from Jiesijie, Co., Ltd (Shanghai, China). The animal experiment protocol was approved by the Animal Care and Use Committee of Ruijin Hospital, Shanghai Jiao Tong University and performed following the National Institutes of Health guidelines, and we tried to relieve their pain as much as possible. Animals had adequate food and water and maintained in separate cages at room temperature under a regular light/dark cycle.

### BM-MSCs isolation and identification

BM-MSCs were separated from Sprague Dawley rats (Jiesijie) weighing 200 - 230 g as previously described [44]. Briefly, remove the muscle and ligament attached to the separated femurs and tibias as far as possible, and then wash the tibia and femoral bone marrow cavity with phosphate buffered saline (PBS) to obtain BM-MCSs [45]. Pure passages from 2 to 5 were applied for the following experiments. And flow cytometric analysis of cell surface markers was used to identify BM-MSCs as described previously. After trypsinized into single cell suspension, stained by first antibodies (anti-rat CD34, CD45, CD29, and CD90) for 30min, then incubated with corresponding secondary FITC antibody under the manufacturer's instructions (Cyagen Biosciences Inc., Guangzhou, China) for another 30min, BM-MSCs were eventually suspended in 300μl PBS for identification by flow cytometry (BD Biosciences, Mississauga, ON).

### ICH models *in vivo* and BM-MSCs labeling and transplantation

ICH models were successfully established by injecting collagenase IV (Sigma-Aldrich, MO, USA) with 0.075U/ 0.4μL PBS into the right basal ganglia in mice as previously reported, briefly, 0.5 mm anterior, 3.5mm ventral, and 2.2mm lateral to the bregma with a rate of 0.1μl/min, as shown in Supplementary Figure 2B. The needle was slowly removed to avoid reflux 5min later. The burr hole was sealed with bone wax, and the incision was sutured. Sham-operated mice underwent the same procedures without the injection of collagenase IV.

PKH26 red fluorescent cell linker mini kit (PKH26, Sigma) was applied for tracking after transplantation. BM-MSCs were trypsinized and resuspended with 2 μM PKH26 dye at room temperature (RT) for 5 min according to the manufacturer’s instructions. Mice were divided into two groups at 24 h after ICH. For the ICH + BM-MSCs treated group, 2×10^6^ BM-MSCs/ 2μl PBS were stereotactically injected into the ipsilateral lesion area with a rate of 0.1μl/min and placed for another 5 min thereafter. For the ICH + PBS treated group, an equal volume of PBS was administrated to the same position.

### ICH models *in vitro* and BM-MSCs coculture

The primary astrocytes were prepared from the pallium of fetal C57BL/6 mice (embryonic days 16-18) (Jiesijie). The isolation and culture of primary astrocytes performed as previously described [4]. Pure passages from 2 to 5 were applied for the following experiments. Astrocytes were incubated into 6/12/24/96-well plates, cocultured with BM-MSCs in the transwell system, exposed to 30μM hemin for 24h to imitating the ICH model *in vitro* for further experiments and detections.

### Experimental design

Mice were random divided into three groups: (1) group 1, sham (n = 45), (2) group 2, ICH + PBS treated (n = 50), and (3) group 3, ICH + BM-MSCs treated (n = 50) group. At 1, 3, 7, 14d following BM-MSCs transplantation, neurological score and behavioral experiments were carried out before mice were sacrificed. Brain samples were collected for further experiments. The experimental schematic diagram *in vivo* is shown in Supplementary Figure 2A.

In the experiments *in vitro*, we explored the underlying mechanism of the role of MST1 and YAP *in vitro*. Primary cultured astrocytes were seeded on 6/12/24/96-well plates, with or without BM-MSCs coculture via a transwell system (3.0μm Pore Size, Corning, USA), under 30μM hemin exposure for 24 h, as shown in Figure 2C. The physiological changes of astrocytes were analyzed according to the proportion of 1: 10 for 24 h of BM-MSCs coculture.

### Brain hematoma volume

Hematoma volume was measured using Cresyl Violet acetate (Sigma) staining. Sections (20μm) were obtained from all brain tissue including the hematoma area. The hematoma area of each section was depicted by image and measured by ImageJ software (National Institutes of Health, Bethesda, MD) as previously described [46]. Then, sum all cerebral hematoma volume of lesion areas by slice thickness.

### Neurobehavioral Evaluation

Behavioral assessments were examined by the modified Neurological Severity Scores (mNSS) and the corner test, performed at 1, 3, 7, 14d after BM-MSCs transplantation, which was performed by two partners who did not know the treatment conditions. The mNSS ranging from 0 to 14 score, consists of response absence (0 - 2), raising mice by the tails (0 - 3), walking on the floor (0 - 3), and beam balance tests (0 - 6). According to the scoring criteria [46], the higher the score, the more serious the injury. For the corner turn test, the mouse was allowed to walk down a corridor into a 30° corner. Mice would turn right or left to exit the corner. The number of right and left turns out of 10 total attempts was recorded. The laterality index (LI) and normalized LI were calculated as previously proposed [47]. The LI was calculated for each mouse, following the formula: LI = (number of right turns - number of left turns)/ (total number of turns). The LI for the day before surgery (LI_BS_) and each of the post-surgery days was calculated, and normalized by the formula: Normalized LI = (LI + 2)/(LI_BS_ + 2).

### siRNA transfection

Hemin (Aladdin, China) was dissolved in absolute ethyl alcohol and diluted with PBS. Astrocytes were transfected with small interfering YAP RNA (si-YAP); Cy3-labeled specific small interfering MST1 RNA (si-MST1) and negative control siRNA (si-NC) (GenePharma, China) by Lipofectamine® 3000 reagent (Invitrogen, USA) under the manufacturer's instructions. The sequences are listed as followed (sense/antisense, 5’-3’), si-YAP: CAGGUGAUACUAUCAACCAAATT/UUUGGUUGAUAGUAUCACCUGTT; si-MST1: GAGAUAUCAAGGCGGGAAATT/UUUCCCGCCUUGAUAUCUCTT; si-NC: UUCUCCGAACGUGUCACGUTT/ACGUGACACGUUCGGAGAATT.

### Cell viability assay and lactate dehydrogenase (LDH) assay

Cell viability and LDH was determined with Cell Counting Kit-8 (CCK-8, Beyotime, China) and LDH cytotoxicity kit (Beyotime) under the manufacturer's instructions. Astrocytes were plated into 96/24-well plate, with or without BM-MSCs coculture, treated with hemin for another 24 h. The absorbance at 450 nm and 490 nm was read with the microplate reader (BioTek, USA).

### Total RNA extraction and quantitative real-time PCR (RT-PCR) analysis of cytokines

Total RNA was extracted from primary astrocytes with Trizol reagent (Invitrogen, USA). Taq DNA polymerase and reverse transcriptase (Yeasen BiotechCo., Ltd., China) were used to reverse transcribed and amplified the total RNA. The expression level of all transcripts was normalized to mRNA of glyceraldehyde 3-phosphate dehydrogenase (GAPDH). The mRNA relative expressions were ultimately normalized to control groups. The primers used to amplify target genes were presented as follows (sense/antisense, 5’-3’): IL-6: TGGGACTGATGCTGGTGACA/ACAGGTCTGTTGGGAGTGGT; IL-10: CTGCTATGCTGCCTGCTCTTACTG/ATGTGGCTCTGGCCGACTGG; TNFα: TGATCGGTCCCAACAAGGA/TGCTTGGTGGTTTGCTACGA; TEAD2: AGGTGGCGGTGGCTTCTATGG/GTAGGCAGTACACAGCAGCAGTTC; GAPDH: GATGGTGAAGGTCGGTGTGA/TGAACTTGCCGTGGGTAGAG.

### Cell proliferation assessment

Astrocytes were seeded onto coverslips with or without BM-MSCs coculture and treated with 30μM hemin for 24 h. In additional experiments, additional si-YAP and si-NC was administered with BM-MSCs and 30 μM hemin for 24 h. Cells were fixed by 4% PFA for 10 min and then subjected to Ki67 (1:500, Abcam) immunofluorescence staining. Fluorescence images were captured by a confocal laser-scanning microscope (Leica, Solms, Germany). Five random fields from each sample were selected to calculate the average percentage of Ki67 positive cells.

### Immunofluorescent staining

Brain cryosections and astrocytes coverslips were immunostained with following primary antibodies: rabbit anti-YAP polyclonal antibody (1:100, Santa Cruz Biotechnology, USA), rabbit anti-Ki67 polyclonal antibody (1:500, Abcam), rabbit anti-GFAP polyclonal antibody (1:1000, Servicebio, China), mouse anti-VIM monoclonal antibody (1:500, Servicebio), mouse anti-GFAP monoclonal antibody (1:500, Servicebio). Secondary antibodies used included Alexa Fluor 488 goat anti-mouse IgG, Alexa Fluor 488 goat anti-rabbit IgG, Alexa Fluor 555 donkey anti-rabbit IgG, Alexa Fluor 555 donkey anti-mouse IgG and, and Alexa Flour 647 goat anti-mouse IgG (1:500, Beyotime). Nuclei were stained with DAPI (1:3000, Beyotime). The fluorescence images were observed and analyzed by confocal laser-scanning microscope (Leica).

### Western blot analysis

Brain tissues and astrocytes were treated according to experiment design. The following primary antibodies were applied in western blot: rabbit anti-YAP polyclonal antibody (1:500, Santa Cruz Biotechnology), rabbit anti-Phospho-YAP polyclonal antibody (1:500, Santa Cruz Biotechnology), rabbit anti-MST1 polyclonal antibody (1:1000, CST), rabbit anti-Phospho-MST1 polyclonal antibody (1:1000, CST), rabbit anti-GFAP polyclonal antibody (1:1000, Servicebio), mouse anti-VIM monoclonal antibody (1:1000, Servicebio), rabbit anti-ALDH1L1 polyclonal antibody (1:1000, Servicebio), rabbit anti-EAAT1 polyclonal antibody (1:1000, CST), rabbit anti-histone-H3 monoclonal antibody (1:1000, Servicebio), and mouse anti-GAPDH monoclonal antibody (1:2000, Servicebio). Enhanced chemiluminescence solution (Thermo Fisher Scientific) and Tanon Image (Shanghai, China) were applied to detect the chemiluminescence signal. The relative intensity of the bands was measured by ImageJ software.

### Statistical analysis

Data were presented as mean ± standard deviation (SD) of at least 3 independent experiments (n). GraphPad Prism 6.0 software (GraphPad, USA) was used for statistical charts and analysis. Statistical comparison was compared by one-way analysis of variance (ANOVA) tests or Student's t-test. A *p*-value less than 0.05 was considered statistical significance.

## Supplementary Material

Supplementary Figures
