# Supplementary material for: Bone marrow mesenchymal stem cells transplantation alleviates brain injury after intracerebral hemorrhage in mice through the Hippo signaling pathway
Source: Aging (Albany NY). 2020 Apr 9;12(7):6306–23. doi: 10.18632/aging.103025 (PMC7185092; doi:10.18632/aging.103025)
Supplement: Supplementary Figures [file aging-12-103025-s001..pdf]

SUPPLEMENTARY FIGURES

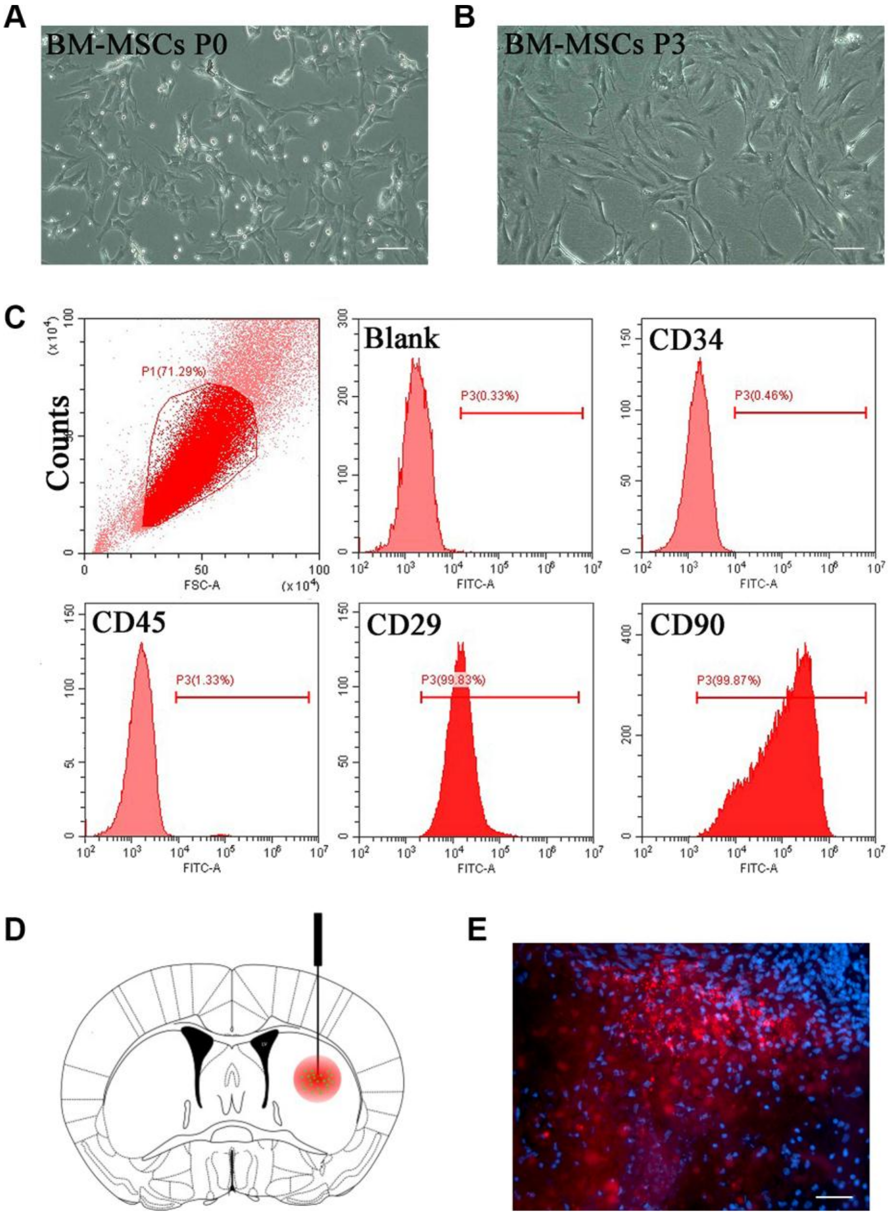

Supplementary Figure 1. BM-MSCs' identification. (**A**, **B**) Morphology of MSCs in cell culture. Cultured cells (passage 0 and passage 3) showed typically spindle-shaped morphology under phase-contrast microscopy. Bar = 50μm. (**C**) Flow cytometry analysis of BM-MSCs at passage 3 depicted that cultured cells were negative for CD34 and CD45, and positive for CD29 and CD90 (n = 4). (**D**) Schematic diagram of BM-MSCs (green) stereotactically injected into the lesion area (red). (**E**) Red fluorescent (PKH26 dye) cells were located in the lesion area after 3 days of injection. Bar = 50μm.

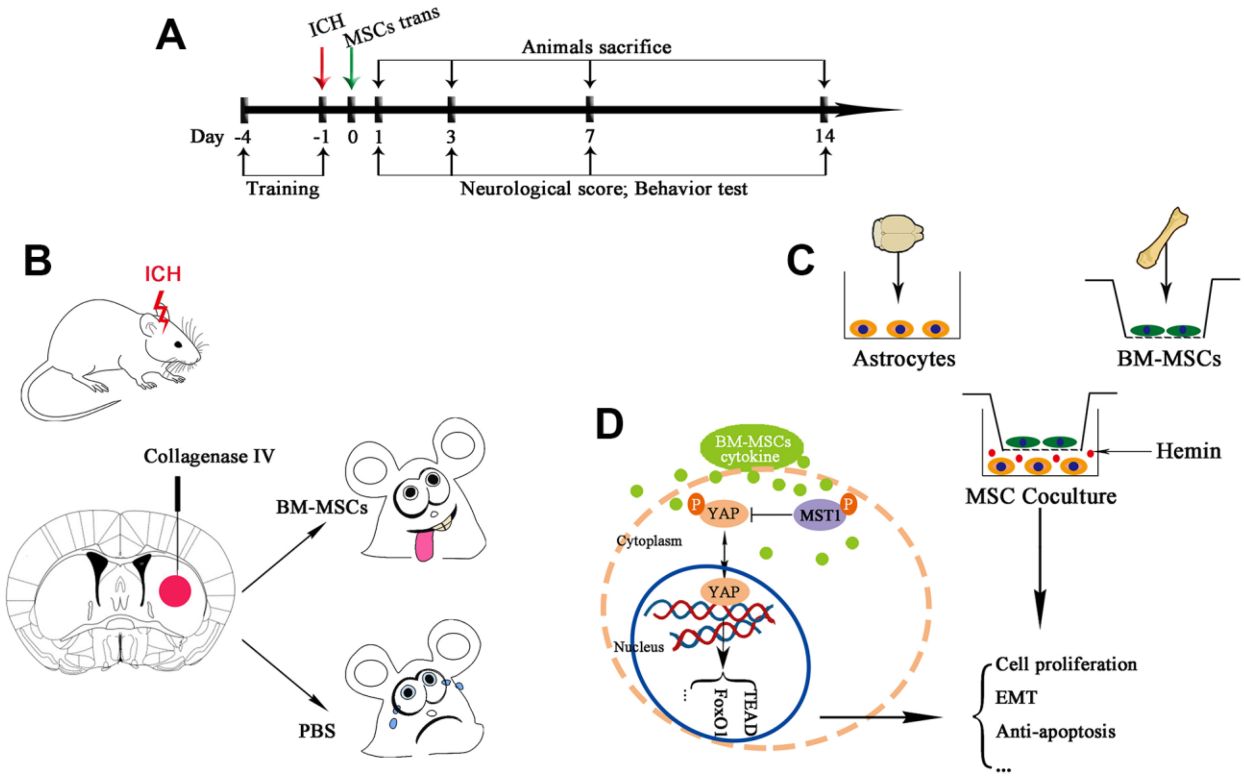

Supplementary Figure 2. Diagram outlining the potential mechanism of BM-MSCs enhancing astrocytes proliferation via MAT1/YAP/Hippo signal pathway. (**A**) The experimental schematic diagram in vivo. (**B**) ICH model in vivo and BM-MSCs transplantation. (**C**) ICH model in vitro. Primary cultured astrocytes were cocultured with or without BM-MSCs via a transwell system. (**D**) Mechanism of BM MSCs inhibiting phosphorylation of MST1 and promoting YAP nuclear translocation.
